# Health and wealth in Mesoamerica: findings from Salud Mesomérica 2015

**DOI:** 10.1186/s12916-015-0393-5

**Published:** 2015-07-14

**Authors:** Ali H. Mokdad, Marielle C. Gagnier, K. Ellicott Colson, Paola Zúñiga-Brenes, Diego Ríos-Zertuche, Annie Haakenstad, Erin B. Palmisano, Brent W. Anderson, Sima S. Desai, Catherine W. Gillespie, Tasha Murphy, Paria Naghavi, Jennifer Nelson, Dharani Ranganathan, Alexandra Schaefer, Gulnoza Usmanova, Shelley Wilson, Bernardo Hernandez, Rafael Lozano, Emma Iriarte

**Affiliations:** Institute for Health Metrics and Evaluation, 2301 5th Ave, Suite 600, Seattle, WA USA; Division of Epidemiology, School of Public Health, University of California, Berkeley, California; Salud Mesoamérica 2015 / Inter-American Development Bank, Calle 50, Edificio Tower Financial Center (Towerbank), Piso 23, Panamá, Panamá; Pardee RAND Graduate School, 1776 Main Street, Santa Monica, California 90401 USA; AARP Public Policy Institute, 601 E Street NW, Washington, DC USA; University of Washington School of Social Work, 4101 15th Avenue N, Seattle, WA USA; George Washington University, 950 New Hampshire Ave, NW, Washington, DC USA

**Keywords:** Maternal and child health, Poverty and health, Health disparities, Central America, Salud Mesomérica 2015

## Abstract

**Background:**

Individual income and poverty are associated with poor health outcomes. The poor face unique challenges related to access, education, financial capacity, environmental effects, and other factors that threaten their health outcomes.

**Methods:**

We examined the variation in the health outcomes and health behaviors among the poorest quintile in eight countries of Mesoamerica using data from the Salud Mesomérica 2015 baseline household surveys. We used multivariable logistic regression to measure the association between delivering a child in a health facility and select household and maternal characteristics, including education and measures of wealth.

**Results:**

Health indicators varied greatly between geographic segments. Controlling for other demographic characteristics, women with at least secondary education were more likely to have an in-facility delivery compared to women who had not attended school (OR: 3.20, 95 % confidence interval [CI]: 2.56-3.99, respectively). Similarly, women from households with the highest expenditure were more likely to deliver in a health facility compared to those from the lowest expenditure households (OR 3.06, 95 % CI: 2.43-3.85). Household assets did not impact these associations. Moreover, we found that commonly-used definitions of poverty do not align with the disparities in health outcomes observed in these communities.

**Conclusions:**

Although poverty measured by expenditure or wealth is associated with health disparities or health outcomes, a composite indicator of health poverty based on coverage is more likely to focus attention on health problems and solutions. Our findings call for the public health community to define poverty by health coverage measures rather than income or wealth. Such a health-poverty metric is more likely to generate attention and mobilize targeted action by the health communities than our current definition of poverty.

## Background

The relationship between poverty and health has been studied in depth from various viewpoints. Numerous studies associate lack of economic development with poor health outcomes [1]. The poor are less likely to seek medical care and more likely to incur catastrophic health spending [2, 3]. Poor women are less likely to seek or receive contraceptives, prenatal care, or skilled birth attendants [2]. Generally, poverty puts households at risk for malnutrition, reduced access to health services, and higher mortality rates.

Many categorizations of the poor employ a uniform definition of poverty. Whether using country-specific income levels, the global $1.25 per day threshold [4], or the Latin America regional $2.50 per day threshold [5], these analyses group all impoverished people into a single homogenous unit, assuming that health is uniform among people slated as poor. However, when it comes to health, huge disparities exist among the 1.22 billion people living below the poverty line in 2010 [6].

With this in mind, a bulk of literature emphasizes the multidimensionality of poverty. As opposed to income or wealth, deprivation, such as lack of access to health care or poor health outcomes, has come into focus [7, 8]. The Bangladesh paradox shows that achieving exceptional progress in key health indicators is possible despite persistent economic poverty [9]. The multidimensional poverty index developed by Alkire and colleagues incorporates three dimensions, including health, to capture progress in reducing deprivation as an alternative to the $1.25 threshold and other income-based poverty assessments [10].

Building on this school of thought, we contend that to improve health outcomes among the poor, a better measure of poor health is needed. The poor face unique challenges related to access, education, financial capacity, environmental effects, and other factors that threaten their health outcomes. Behaviors related to delivery care are especially sensitive to social and economic conditions, particularly in areas where quality of care and culturally relevant practices are important [11, 12]. However, a health index to assess disparities and call for action would generate more interest among the health communities. Indeed, the Millennium Development Goals (MDGs) have raised health awareness and initiated programs and foreign aid to tackle health problems [13]. Building on this success and creating a health poverty index would be ideal.

In this article we examine the association of health outcomes with several household indicators among poor populations. Notably, it harnesses data collected across poor populations in Central American countries as part of the Salud Mesoamérica 2015 Initiative (SM2015), a regional action aimed at improving the health conditions of the poor. We examine the need for a health index to measure “health wealth” as a predictor of health outcomes while adjusting for health behaviors and known confounders among the poorest quintile living in eight countries of Mesoamerica.

## Methods

### Study design and participants

The data presented were collected as part of the baseline evaluation for SM2015, which was established to address the health issues faced by the poorest quintile of the population in El Salvador, Guatemala, Honduras, Nicaragua, Belize, Costa Rica, Panama, and Mexico. Surveys were conducted in households and health facilities in each country. We conducted our own censuses within each selected primary sampling unit, a segment of approximately 150 households, in order to identify eligible households. This ensured we used the correct denominator in indicator estimation and allowed us to account for the potential movement of the population in the study areas since the last national census. Among eligible households, a randomly selected subset was chosen for the household survey.

The household survey had three components. A household questionnaire captured information on assets, wealth, and characteristics of the home. A maternal health questionnaire collected demographic, health behavior and reproductive health information on women of reproductive age (15–49 years). A child health questionnaire on health, diet, and vaccination history was completed for children 0- to 59-months old. Physical measurements and anemia tests were conducted for children.

To assess maternal education, women were asked if they have ever attended school and if they have ever completed a literacy course. Women who responded that they have attended school were asked about the highest level of schooling that they attained: primary (elementary school), secondary (middle school), preparatory or university. To assess reproductive health indicators, women were asked to answer questions about their birth history in the last five years. For each birth, women were asked if they had received at least one antenatal care (ANC) visit. Women who had received at least one ANC visit were further asked about the number of visits attended. For each ANC visit, women were asked to indicate the person who provided them with care. Interviewers were instructed to have women specify the most qualified attendant during each of these visits. To assess skilled birth attendance (SBA) and in-facility delivery, women were asked to identify each person who provided them with attention during birth and to indicate where they gave birth. Women were also asked if they used any family planning method after each birth. Women who had used family planning, were asked what method was used and how soon after birth did they start using this method.

To assess post-natal care for each child in the last five years, women were asked if the child was examined by a health provider at some time after birth and to indicate how many hours, days, or weeks after birth the child had a first post-natal care. In reference to each child, women were then asked if they breastfed at least one time. To assess early initiation, women were asked how soon after birth they breastfed for the first time. To assess exclusive breastfeeding, questions were asked about a 24-hour dietary recall for each child born in the six months prior to the date of the survey.

To assess immunization coverage, interviewers reviewed child vaccination cards and recorded the vaccines and dates marked on the cards for each child under five years old. Vaccination recall was assessed by asking women to indicate all of the vaccines that each child had received. Questions about vaccines were asked in adherence to national vaccination schemes for each country.

The SM2015 surveys were conducted using a computer‐assisted personal interview (CAPI) by trained interviewers. Data were continuously monitored by the Institute for Health Metrics and Evaluation (IHME). All data were collected after obtaining informed consent. The field surveyors explained the purpose of this study to participants. Then, written informed consent was obtained from all study participants who agreed to participate prior to data collection. The study received institutional review board (IRB) approval from the University of Washington, partnering data collection agencies, and the Ministry of Health in each country to ensure that the data were collected in an appropriate and ethical manner. Baseline surveys were conducted from 1 March 2011 to 31 August 2013. We used Stata 12.1 and Stata 13.1 for the analyses. All estimates are computed using survey weights, unless otherwise noted. Additional details on SM2015 design, sampling, methodology, and implementation are available elsewhere [14].

### Definitions

Household monthly expenditure was computed as the sum of reported weekly, monthly, or semi-annual expenditures after being converted to monthly totals: food, alcohol and tobacco, education-related expenses, household utilities; clothing and footwear, transportation, communication, out-of-pocket health care costs, social security premiums, private insurance premiums, and associated health care costs. Households that spent 25 % or more on health care were considered to have incurred catastrophic health expenditure in the past month.

Key child health indicators were also computed. Adherence to national vaccination schemes for all vaccines and for measles, mumps, and rubella (MMR) were estimated based on caregiver recall and vaccination card information. Anthropometric measurements of children were used to calculate the prevalence of wasting and stunting, defined as −2 standard deviations below the mean height-for-age and weight-for-height according to World Health Organization (WHO) criteria, respectively [15]. Additionally, we assessed whether children with signs of diarrhea in the past two weeks received proper oral rehydration salt (ORS) treatments.

Reproductive health indicators included services received during the antenatal period, delivery care, and breastfeeding. Among deliveries in the two years prior to the survey, we estimated coverage of ANC and SBA with a doctor or nurse. We focused on SBA and in-facility deliveries as they are strongly associated with a reduction in maternal and infant mortality [16]. Exclusive breastfeeding during the first six months of life was estimated using a 24-hour dietary recall; all children 0- to 5-months old who consumed exclusively breast milk, as reported by a caregiver, were considered adherent.

A sample of mother-child pairs was used to compute a composite coverage score of select maternal, newborn, and child health indicators. Data were linked for each child, mother, and corresponding birth history and restricted to each woman’s youngest child born in the two years prior to the survey. This score is equal to the summed presence of eight select health indicators: one ANC visit with a skilled attendant, four ANC visits with a skilled attendant, SBA, use of ORS treatment for recent diarrhea, initiation of breastfeeding within 24 hours of birth, complete childhood vaccination based on age and national scheme, absence of stunting, and absence of wasting. Segment-level coverage was computed for each subcomponent in order to calculate correlation with segment average wealth. The highest possible score is 8 and was converted to a proportion for some analyses. For each mother/child pair we present the health indicators as a continuum of care (ANC1, ANC4, SBA, in-facility delivery, breastfeeding initiation within one hour, skilled post-natal care for baby within one week, use of modern contraceptive, and complete immunization) by education and expenditure.

### Statistical analyses

The surveys were conducted in communities that were designated as the poorest areas in each country. Even so, substantial income variability was found within these communities, with monthly household expenditure per month ranging from USD 3 to 1,200 per month. In order to examine the variation of wealth and health indicators within our sample, we computed prevalence and uptake of select indicators at the segment-, municipality-, and country-level. In addition, the sample was stratified by maternal education level and by household expenditure quintile to examine variation by country in the uptake of select health indicators in the continuum of maternal and child care. Average uptake of health-seeking behaviors from prenatal through early childhood care is reported for each subgroup.

We used multivariable logistic regression to measure the association between delivering in a health facility and select household and maternal characteristics. Data for each mother-child pair were pooled across countries, and a country-specific fixed effect was added to account for different patterns of SBA between countries. Model 1 covariates include within-country household expenditure quintile, asset index, attained maternal education level (no education, primary education, or secondary or higher education), maternal age in years at the time of the survey, and maternal parity. An alternative asset-based metric of wealth was computed as a factor score and the results are similar (data available upon request). Model 2 incorporated additional covariates of household characteristics and women’s autonomy, including household size, head of household gender, maternal occupational status, and maternal marital status. Model 3 included information on potential barriers to care: whether the mother is insured, travel time to the usual health facility, whether the mother received counseling from a community health worker during the past three months, whether the mother was exposed to media (newspaper, radio, or television) during the past week, and mother-reported barriers to care. If travel time to the usual health facility is missing, we used the travel time to the closest health facility. If that too was missing, we used the median travel time to the usual facility among households in that segment. Barriers to care were reported among women who had a recent illness but did not seek medical care, so dummy variables were added to reflect women who were not recently ill or were ill but did seek care.

Less than 7 % of observations were excluded from the regression analysis because they lacked information about one or more of the independent variables. We used self-reported barriers to care in our model to account for potential bottlenecks from the women’s side in seeking required health care. Alternative models using a dependent variable of SBA and in-facility delivery with SBA were also conducted, but the results were similar (data available upon request). In order to assess whether health seeking patterns varied by country in relation to health insurance, we tested for interaction between country and health insurance in our models. We found no statistically significant interaction between the two variables.

### Role of the funding source

The funders of this study had no role in study design, data collection, data analysis, interpretation, or writing of the report. The corresponding author had full access to all the data in the study and had final responsibility for the decision to submit for publication.

## Results

In total, out of the 11,685 segments we conducted 90,000 censuses and completed interviews in 20,225 households in El Salvador (523;14,230; 3,625), Guatemala (1,033;20,438;4,420), Honduras (353;15,726;2,971), Mexico (8,162;24,343;5,428), Nicaragua (1,455;8,864;2,071), and Panama (158;4,945;1,710) (Table 1). We conducted interviews in 716 segments with an average of about 28 households, 37 women, and 32 children interviewed per segment.

**Table 1:** Sample description by country

Country	SM2015 municipalities	Segments	Selected segments	Households censused	Households surveyed	Women surveyed	Children surveyed
El Salvador	14	523	136	14230	3625	4730	3328
Guatemala	27	1033	148	20438	4420	5899	5404
Honduras	35	353	99	15726	2971	3580	3192
Mexico	56	8163	181	24343	5428	6988	6499
Nicaragua	23	1455	91	8864	2071	2823	2236
Panama	2	158	61	4945	1710	2453	2253
Total	157	11685	716	88546	20225	26824	23223

All *N*

*SM2015* Salud Mesoamerica 2015

There was substantial variation in monthly household and per capita expenditure among the segments within the study area, indicating wide disparities in these impoverished areas (Table 2). Catastrophic health expenditure ranged from 5.5 % of all households in Guatemala to 19.0 % of all households in Honduras. However, in some segments, these levels reached more than 60 % of households (63.3 % in Honduras) or even 100 % of households (in Panama). The highest average composite coverage scores, indicating the highest uptake of health interventions, were in El Salvador (average of 5.6) and Nicaragua (5.5), and the lowest average score was in Guatemala (2.8).

**Table 2:** Variation in household wealth, catastrophic health expenditure, and health score by country

Country	Average monthly household expenditure in USD^a^ (LCU)	Monthly expenditure per capita in USD (LCU)	Lowest monthly household expenditure in USD (LCU)		Average % of monthly household expenditure for healthcare	Highest average % of monthly household expenditure for healthcare		% household with catastrophic health expenditure^b^	Highest % household with catastrophic health expenditure		Average health score^c^	Lowest average health score	
			Municip	Seg		Municip	Seg		Municip	Seg		Municip	Seg
El Salvador	167 (167)	46 (46)	123 (123)	74 (74)	5.4	8.7	20.9	7.4	11.3	33.3	5.6	5.3	4.3
Guatemala	157.4 (1,237)	43.6 (343)	89.3 (702)	46.3 (364)	3.6	8.7	15.3	5.5	16.7	30.0	2.8	1.6	0.8
Honduras	202.8 (3,837)	42.8 (809)	104.5 (1,978)	69.3 (1,312)	3.9	11.4	11.4	19.0	45.5	63.3	4.9	3.7	3.2
Mexico	187.5 (2,395)	40.3 (515)	77.6 (991)	58.7 (750)	4.7	10.6	14.4	11.9	37.6	50.0	3.9	1.9	1.6
Nicaragua	202.3 (5,001)	44.2 (1,092)	105.9 (2,617)	57.0 (1,408)	3.0	6.7	7.9	9.4	20.0	28.6	5.5	4.7	4.4
Panama^d^	254 (254)	53 (53)	225 (225)	44 (44)	2.4	2.6	11.2	13.2	18.1	100	3.4	3.4	1.0

*ANC* antenatal care, *Municip* municipality, *ORS* oral rehydration salts, *Seg* Segment, *LCU* local currency unit

^a^Based on World Bank official exchange rate, 2009–2013 period average, except for Honduras (2011 rate)

^b^Catastrophic health expenditure defined as >25 % of monthly household expenditure on health care

^c^Health score ranges from zero to eight, indicating achievement of: one ANC visit with a skilled attendant, four ANC visits with a skilled attendant, delivery with a skilled birth attendant, use of ORS treatments for recent diarrhea, initiation of breastfeeding within 24 hours of birth, complete childhood vaccination based on age, absence of stunting, absence of wasting

^d^In Panama, these are provinces, not municipalities

Health indicators varied greatly between municipalities and segments (Table 3). For example, in Guatemala the MMR immunization rate was 88.4 %, with a range from 73.2 % to 94.3 % among municipalities and from 38.2 % to 100 % among segments. Wasting reached 100 % in certain segments of Mexico, while there was no wasting in at least one segment of each country. In El Salvador, SBA ranged from 67.5 % to 100 % for municipalities and 20 % to 100 % for segments, while the overall average for the six countries studied was 85.5 % (Table 4). Large variations exist in the practice of exclusive breastfeeding for all countries, with segment-level prevalence ranging from 0 to 100 %.

**Table 3:** Variation in indicators of child health by country

Country	MMR immunization^a^ (%)			Complete immunization^b,c^ (%)			Wasting^b^ (%)		
	Overall	Range across mun.	Range across segments	Overall	Range across mun.	Range across segments	Overall	Range across mun.	Range across segments
El Salvador	78.5	73.2 - 86.4	47.4 - 100	70.0	64.2 - 84.0	33.3 - 100	2.4	0 - 4.2	0 - 28.6
Guatemala	88.4	72.0 - 94.3	38.2 - 100	16.0	0 - 50.0	0 - 60.0	1.6	0 - 4.0	0 - 13.3
Honduras	95.1	88.7 - 100	74.1 - 100	40.0	4.9 - 64.3	0 - 69.7	1.5	0 - 4.0	0 - 10.0
Mexico	79.5	33.7 - 100	32.7 - 100	40.2	9.8 - 90.2	0 - 100	1.4	0 - 5.4	0 - 100
Nicaragua	92.9	70.4 - 97.8	64.0 - 100	49.6	26.9 - 62.0	17.6 - 75.8	1.5	0 - 4.7	0 - 10.3
Panama^e^	90.5	89.5 - 90.8	50.0 - 100	18.0	10.9 - 20.5	0 - 48.4	2.8	1.8 - 3.2	0 - 16.7
	Stunting^b^ (%)			ORS coverage^b,d^ (%)					
	Overall	Range across mun.	Range across segments	Overall	Range across mun.	Range across segments			
El Salvador	16.3	2.2 - 34.6	0 - 57.6	64.2	0 - 91.8	0 - 100			
Guatemala	59.2	21.5 - 79.9	11.1 - 86.0	60.9	0 – 100	0 - 100			
Honduras	22.2	3.4 - 53.0	0 - 66.7	54.8	0 – 100	0 - 100			
Mexico	36.9	3.4 - 74.1	0 - 80.0	50.5	0 – 100	0 - 100			
Nicaragua	14.0	0 - 28.0	0 - 33.3	53.9	9.0 - 100	0 - 100			
Panama^e^	55.9	33.0 - 63.9	0 - 83.5	57.5	57.4 - 57.7	0 - 100			

*Mun*. municipalities, *ORS* oral rehydration salts

^a^MMR: measles, mumps, rubella (1 dose); children 12–59 months

^b^Children 0–59 months

^c^Complete immunization for age according to national immunization scheme

^d^Coverage of ORS among children with symptoms of diarrhea in the past two weeks

^e^In Panama, these are provinces, not municipalities

**Table 4:** Variation in indicators of maternal and newborn health by country

Country	Antenatal care			Antenatal care			Skilled birth			Exclusive		
	(one visit, %)^a^			(four visits %)^a^			attendance (%)^a^			breastfeeding (%)^b^		
	Overall	Range across mun.	Range across segments	Overall	Range across mun.	Range across segments	Overall	Range across mun.	Range across segments	Overall	Range across mun.	Range across segments
El Salvador	96.8	93.5 - 100	80.0 - 100	90.0	84.2 - 100	40.0 - 100	85.5	67.5 - 100	20.0 - 100	60.5	0 - 78.0	0 - 100
Guatemala	30.4	7.1 - 71.4	0 - 92.3	25.3	3.8 - 83.3	0 - 90.0	24.7	2.1 - 71.4	0 - 100	79.5	27.5 - 100	0 - 100
Honduras	83.6	48.8 - 100	20.0 - 100	69.6	32.5 - 100	15.4 - 100	81.1	51.3 - 100	16.7 - 100	47.3	0 - 100	0 - 100
Mexico	74.1	27.0 - 100	0 - 100	57.9	10.5 - 90.0	0 - 100	46.3	0 -100	0 - 100	55.3	0 - 100	0 - 100
Nicaragua	95.3	88.7 - 100	57.1 - 100	80.4	73.3 - 91.8	40.8 - 100	89.8	46.5 - 100	37.5 - 100	55.6	0 - 100	0 - 100
Panama^c^	78.0	71.6 - 80.0	0 - 100	38.1	34.8 - 49.0	0 - 87.0	70.2	49.4 - 76.6	0 - 100	45.3	43.1 - 46.0	0 - 100

*Mun*. municipalities

^a^Most recent pregnancy in last two years

^b^Children 0–5 months old

^c^In Panama, these are provinces, not municipalities

**Fig. 1:**
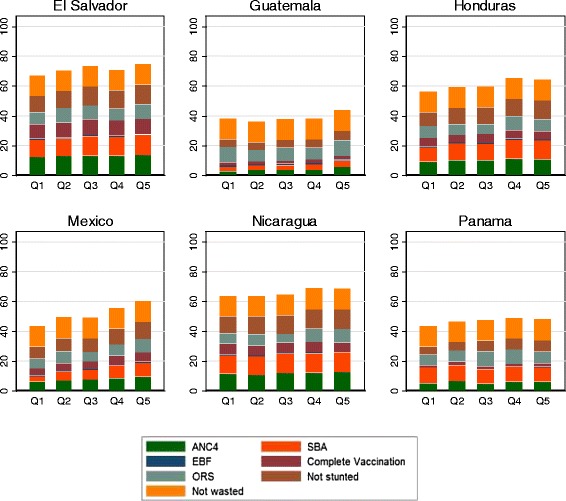
Composite coverage by household expenditure quintile. *ANC4* Antenatal care (four visits). *SBA* Skilled birth attendance. *EBF*: exclusive breastfeeding, *ORS* oral rehydration solution for diarrhea treatment

Figure 1 shows the composite coverage of seven select maternal, newborn, and child health indicators by expenditure quintile for mother-child pairs in the sample (ANC1 was included in ANC4). Indeed, it shows the percentage of women/children receiving each and all the desired interventions. El Salvador exhibits the highest composite coverage across wealth quintiles. Guatemala performs the worst, with composite coverage below 40 % for all but the highest expenditure quintile. In all countries, mother-child pairs in the lowest expenditure quintile have lower composite coverage than those in the highest expenditure quintile. Antenatal and delivery care practices show a higher uptake among the higher expenditure quintiles in most countries, with the exception of Panama and Nicaragua. Stunting is less prevalent among the higher than lower expenditure quintiles.

**Fig. 2:**
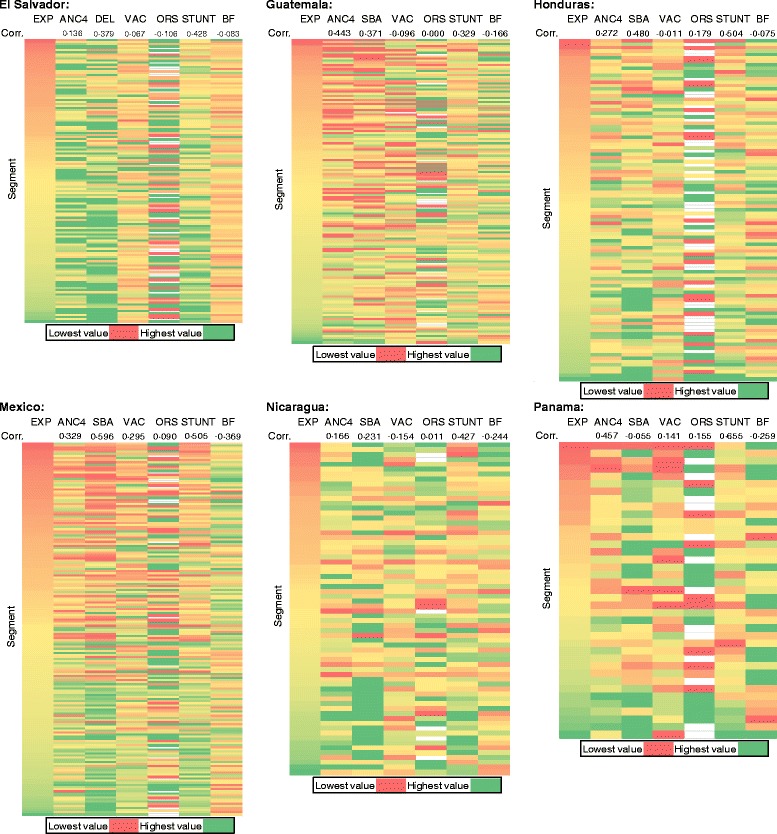
Heatmaps of key indicators of health behavior across segments. *EXP* monthly household expenditure, *ANC4* antenatal care (four visits), *BF* early initiation of breastfeeding, *ORS* oral rehydration solution for diarrhea treatment (White cells for ORS indicate that there were no children exhibiting symptoms of diarrhea in the past two weeks in that segment.), *SBA* skilled birth attendance, *STUNT* percent of children not stunted, *VAC* complete vaccination for age. Correlation is reported for each indicator with household expenditure at the segment level

Figure 2 shows heat maps sorted by expenditure deciles against deciles of several health indicators. Some indicators, including ORS coverage, show no association with wealth (correlation less than positive or negative 0.2). However, SBA and prevalence of stunting are more highly correlated in most countries, particularly Honduras and Mexico (correlation approximately 0.5).

**Fig. 3:**
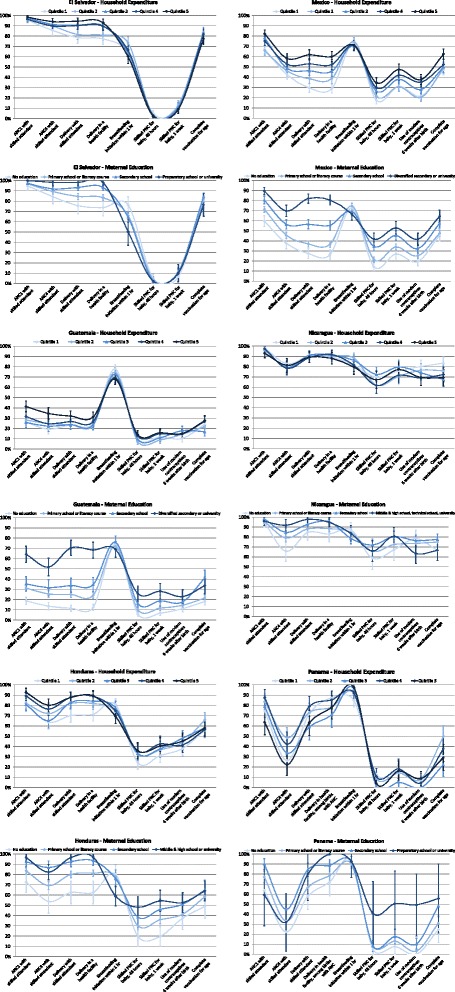
Continuum of care for mother-child pairs, most recent birth in the past two years, by household expenditure quintile or attained maternal education level. *PNC* postnatal care, *ANC1* 1 visit of antenatal care, *ANC*4 4 visits of antenatal care

Figure 3 shows the continuum of care for each woman and her child by household expenditure and education. Stratification by maternal education showed a markedly wider variation in health indicator performance compared to stratification by household expenditure, though the patterns were similar. Less-educated women and their children were less likely to receive health care, particularly services related to delivery. Less-educated women and women from households with lower expenditure adhered better to recommended breastfeeding practices. There is a low reported uptake of in-facility postnatal care for babies among all countries, particularly as compared to antenatal care.

In-facility delivery during the past five years was positively associated with both education and expenditure (Table 5). In our first model, adjusting for country, household expenditure, and maternal age and parity, women with primary education and women with at least secondary education were more likely to have an in-facility delivery compared to women who had not attended school (OR: 1.61, 95 % confidence interval [CI]: 1.35-1.92 and OR: 3.20, 95 % CI: 2.56-3.99, respectively). Similarly, women from households with the highest expenditure were also more likely to have in-facility delivery compared to those from the lowest-expenditure households (OR 3.06, 95 % CI: 2.43-3.85). In Model 2, we added household size, gender of the head of household, occupation, and marital status to our model to account for a woman’s role in the household; both education and expenditure remained strongly associated with in-facility delivery. When we added potential barriers to care to our model (Model 3), both education and expenditure remained significant. Insured women were more likely to deliver in a facility (OR: 1.77, 95 % CI: 1.40-2.24). Women receiving recent counseling from a community health worker were less likely to delivery in a facility (OR: 0.72, 95 % CI: 0.59-0.89). There were independent effects of education and poverty on in-facility delivery, indicating that each is a major contributing factor. Household assets did not impact these associations.

**Table 5:** Association of in-facility delivery with maternal characteristics, household characteristics, intervention exposures, and barriers to care^a^

Characteristic	Model 1		Model 2		Model 3	
	(N = 15,563)		(N = 15,494)		(N = 14,904)	
	Pseudo-R^2^ = 0.297		Pseudo-R^2^ = 0.301		Pseudo-R^2^ = 0.306	
	OR (95 % CI)	p-value	OR (95 % CI)	p-value	OR (95 % CI)	p-value
Country						
GTM	1.00 (ref)		1.00 (ref)		1.00 (ref)	
HND	15.58 (11.37-21.37)	<0.001	15.13 (11.08-20.64)	<0.001	16.36 (11.83-22.61)	<0.001
MEX	3.17 (2.40-4.18)	<0.001	3.17 (2.40-4.18)	<0.001	2.17 (1.58-2.98)	<0.001
NIC	28.53 (20.00-40.69)	<0.001	27.42 (19.20-39.17)	<0.001	28.09 (19.59-40.29)	<0.001
PAN	16.59 (10.12-27.20)	<0.001	19.27 (11.34-32.73)	<0.001	20.07 (11.73-34.35)	<0.001
SLV	19.53 (14.58-26.17)	<0.001	17.64 (12.76-24.38)	<0.001	20.42 (14.75-28.28)	<0.001
Household expenditure						
Quintile 1	1.00 (ref)		1.00 (ref)		1.00 (ref)	
Quintile 2	1.30 (1.07-1.58)	0.009	1.31 (1.07-1.60)	0.010	1.31 (1.07-1.61)	0.010
Quintile 3	1.59 (1.28-1.97)	<0.001	1.59 (1.28-1.99)	<0.001	1.53 (1.22-1.91)	<0.001
Quintile 4	2.39 (1.92-2.99)	<0.001	2.38 (1.90-2.99)	<0.001	2.24 (1.78-2.83)	<0.001
Quintile 5	3.06 (2.43-3.85)	<0.001	2.98 (2.37-3.77)	<0.001	2.82 (2.23-3.56)	<0.001
Asset score	3.74 (1.91-7.33)	<0.001	3.81 (1.88-7.72)	<0.001	2.41 (1.18-4.92)	0.016
Attained education level						
No schooling	1.00 (ref)		1.00 (ref)		1.00 (ref)	
Primary school or literacy course	1.61 (1.35-1.92)	<0.001	1.58 (1.34-1.88)	<0.001	1.46 (1.22-1.75)	<0.001
Secondary school or higher	3.20 (2.56-3.99)	<0.001	2.98 (2.39-3.72)	<0.001	2.64 (2.11-3.31)	<0.001
Age (years)						
15-19	1.00 (ref)		1.00 (ref)		1.00 (ref)	
20-24	1.18 (0.97-1.43)	0.097	1.11 (0.91-1.37)	0.294	1.10 (0.89-1.35)	0.392
25-29	1.49 (1.22-1.82)	<0.001	1.33 (1.08-1.64)	0.007	1.28 (1.03-1.58)	0.025
30-34	2.38 (1.84-3.07)	<0.001	2.08 (1.61-2.70)	<0.001	1.99 (1.53-2.59)	<0.001
35-39	3.76 (2.78-5.09)	<0.001	3.18 (2.36-4.29)	<0.001	3.05 (2.23-4.17)	<0.001
40-44	3.41 (2.35-4.96)	<0.001	2.83 (1.94-4.13)	<0.001	2.62 (1.78-3.84)	<0.001
45-49	4.81 (2.62-8.83)	<0.001	3.79 (2.05-7.02)	<0.001	3.46 (1.85-6.50)	<0.001
Parity	0.72 (0.69-0.76)	<0.001	0.75 (0.71-0.78)	<0.001	0.75 (0.72-0.79)	<0.001
Household size			0.96 (0.92-1.00)	0.043	0.96 (0.92-1.00)	0.069
Head of household is female			1.29 (1.01-1.64)	0.038	1.24 (0.96-1.60)	0.093
Occupation						
Employed and working for money			1.00 (ref)		1.00 (ref)	
Homemaker			0.71 (0.53-0.95)	0.023	0.73 (0.54-0.98)	0.037
Other^b^			1.18 (0.75-1.88)	0.472	1.25 (0.79-2.00)	0.341
Marital status						
Single			1.00 (ref)		1.00 (ref)	
Married			1.25 (0.94-1.65)	0.127	1.23 (0.91-1.64)	0.173
Union			0.97 (0.75-1.24)	0.780	0.95 (0.73-1.23)	0.674
Divorced, separated, widowed, other			0.93 (0.68-1.26)	0.625	0.97 (0.71-1.32)	0.839
Insured					1.77 (1.40-2.24)	<0.001
Travel time to usual health facility^c^					1.00 (1.00-1.00)	0.869
Received counseling from a community health worker in the past month					0.72 (0.59-0.89)	0.002
Exposure to any media in the past week (newspaper, radio, television)					1.31 (1.11-1.54)	0.001
Barriers to seeking medical care						
Mother had no recent illness					1.15 (0.86-1.53)	0.348
Care sought for mother’s recent illness					1.27 (0.90-1.79)	0.180
Among mothers who were recently ill and did not seek care						
Care is too expensive					1.42 (0.77-2.60)	0.258
Health center is too far away					1.10 (0.53-2.28)	0.788
Could not get transportation					0.69 (0.38-1.24)	0.210
Facility infrastructure is poor					2.72 (1.01-7.30)	0.048
Facility has insufficient drugs					1.09 (0.72-1.65)	0.676
Health center is not well equipped					0.59 (0.30-1.19)	0.139
Staff are difficult to deal with					1.67 (0.84-3.34)	0.146
Staff is not knowledgeable					2.38 (0.59-9.58)	0.224
No trust in staff					0.71 (0.32-1.61)	0.417
Could not get permission to go to the doctor					0.59 (0.03-10.85)	0.720
Did not want to go alone					0.76 (0.28-2.12)	0.606
Too busy with work or children					0.69 (0.42-1.16)	0.164

*CI* confidence interval, *GTM* Guatemala, *HND* Honduras, *MEX* Mexico, *NIC* Nicaragua, *OR* odds ratio, *PAN* Panama, *SLV* El Salvador

^a^Models are adjusted for all covariates listed in the corresponding column. All estimates are survey-weighted. Pseudo-R^2^ is based on the unweighted logistic regression model fit

^b^This includes women who reported being employed but not working during the week prior to the survey, a current student, retired, disabled, or unemployed

^c^If travel time to the usual health facility was missing, we used first the travel time to the closest facility; if that was also missing we used the median travel time to the usual health facility for the segment

## Discussion

To our knowledge, this is the largest study conducted in the poorest areas of Mesoamerica. The concentration of these surveys in the poorest areas allowed us to uncover disparities between and within countries in terms of exposure to health interventions, health behaviors, and risk factors among the poor. We found that commonly used definitions of poverty, namely expenditure, do not align with the disparities in health outcomes observed in these communities.

Our findings also underline that empowering women through education is crucial to improving health. Education has a strong association with infant health [17]. Educated women are more likely to understand the signs of health danger, seek medical care, and adhere to the health message provided. This is shown by our results and by anecdotal evidence in the field. During our survey, data collection in one of the countries was halted by community elders who did not approve of our questions about contraception. However, interviews already conducted in this community indicated women were very receptive to the questions. Once we showed the elders the interviews we had already conducted, they remained hesitant, but were ultimately supportive. Local data are powerful, even when dealing with sensitive health topics and issues.

Our study has limitations that have to be considered in the interpretation of results. We used household expenditure instead of household assets in our analyses to examine the availability of cash flow. While wealth as measured by assets that could be converted to cash is a better indicator of total wealth, these assets are not easily liquidated. Using expenditure allows us to gauge how immediately households can respond to their health expenditure needs. We conducted our study in only poor areas; however, the same health index would apply in richer communities as the health indicators we used are set for the whole country. Finally, we used self-reported variables that may be subject to reporting bias and social desirability. However, our study is based on a large sample size and used the same methodology in all countries.

Our use of segments (a unit of approximately 150 households) as a unit of analysis may not provide adequate sample size for drawing conclusions. However, since we conducted our own censuses, our small sample in each segment is representative of that segment. Irrespective of statistical power, we are able to show pockets of need by small geographic units. Moreover, our census within each of our selected segments enabled us to obtain better estimates for total need for services in each area.

The variation in performance within poor areas highlights the need for a more thorough examination of the relationship between poverty and health. These findings call on the public health community to rethink standard definitions of poverty and to examine alternate measures of health in poor areas. We propose using a composite measure of “health poverty” as an alternate way of assessing improvements in the poor’s lives, as opposed to an expenditure- or wealth-based metric. This composite indicator makes comparisons across and within countries easier, as it is not subject to the same currency, purchasing power parity, or other cost-of-living adjustments that make income and wealth measures less reliable for cross-country comparisons. Furthermore, evidence shows that addressing the population’s health problems contributes to reducing poverty as measured by income [18, 19]. Health poverty better encapsulates the poor’s ability to realize their capabilities, as it captures lack of access and other hindrances to enjoying the longest and healthiest life possible.

Health poverty indices should be developed for all aspects of health, from chronic to infectious diseases, classified by health topic. For example, for maternal and child health, the index should measure whether the recommended levels of ANC, SBA at delivery, child immunization, and other relevant indicators are met. Effective coverage, the fraction of potential health gain that is actually delivered to the population through the health system, should also be included when available. The public health community should work on creating such an index, building on the success of the MDGs. Currently, the definitions used most frequently for social determinants of health, such as poverty based on income or education level, are not factors that can be affected by the action of health authorities. It is time for the public health community to own a definition of poverty and be held accountable for it.

We strongly feel that a health index would be better for targeting health programs. It will enable authorities to develop, implement, and evaluate programs. Moreover, this index will capture health disparities better than income, education, or expenditure. Furthermore, it will empower health authorities to act upon health poverty and rally support from governments and donors. A health minister cannot ignore such a bad health index in his/her community, but may not be as motivated or supported by government and donors to eradicate poverty or increase education.

## Conclusions

We believe that the best way to eliminate poverty is by tackling poor health performance. A health index pinpoints the groups and areas that require concerted attention, thereby mobilizing the international community to concentrate on efforts that can truly improve the lives of the poor. A composite indicator of health poverty based on coverage will focus attention on health problems and solutions. Based on this health index, governments can be held accountable more readily for the health of their population, particularly those most in need of health care. Better health is tied to better education and leads to better economies. These results underline how health should be on the frontlines of any effort working to eradicate poverty.

## Abbreviations

ANC: Antenatal care
CAPI: Computer‐assisted personal interview
CI: Confidence interval
IRB: Institutional review board
MDGs: Millenium Development Goals
MMR: Measles, mumps, and rubella
OR: Odds ratio
ORS: Oral rehydration salt
SBA: Skilled birth attendance
SM2015: Salud Mesoamérica 2015 (The Mesoamerican Health Initiative 2015)
WHO: World Health Organization
